# Optimization of the Microbial–Enzymatic Synergistic Treatment of Cottonseed Protein and Evaluation of the Nutritional Value and Antioxidant Activity of Cottonseed Peptides

**DOI:** 10.3390/foods15111902

**Published:** 2026-05-28

**Authors:** Weidong Niu, Changzhao Jin, Hao Liu, Changjiang Zang, Kailun Yang, Yong Chen, Jiancheng Liu

**Affiliations:** Research Center for Biological Feed and Animal Gut Health, College of Animal Science, Xinjiang Agricultural University, Urumqi 830052, China; nwd163com@163.com (W.N.); j17799683492@163.com (C.J.); lh1735469814@163.com (H.L.); zcj780@xjau.edu.cn (C.Z.); yangkailun2002@aliyun.com (K.Y.); cy@xjau.edu.cn (Y.C.)

**Keywords:** microbial–enzymatic synergy, cottonseed protein, response surface methodology, process optimization, cottonseed peptide, antioxidant activity

## Abstract

This study was conducted to optimize the conditions for the synergistic treatment of cottonseed protein with microorganisms and enzymes and to evaluate the nutritional value and antioxidant activity of the resulting cottonseed peptides, with the ultimate goal of improving the nutritional quality of cottonseed protein. In single-factor experiments, laccase, alkaline protease, *Saccharomyces cerevisiae*, and *Lactobacillus acidophilus* were individually applied to cottonseed protein, and the optimal ranges for additive dosage, temperature, moisture content, and treatment duration were established using free gossypol, acid-soluble protein, and pH as response indicators. A Box–Behnken response surface design was subsequently adopted to perform an integrated analysis of the three responses and to determine the optimal conditions for the combined microbial–enzymatic treatment. The nutritional value and antioxidant activity of the cottonseed peptides obtained under these conditions were then evaluated. The optimal process parameters were identified as follows: microbial and enzyme dosages each at 1% (*w*/*w*), temperature of 37 °C, 37% moisture content, and treatment time of 96 h. Under the optimized conditions, the free gossypol content of the treated cottonseed protein was reduced to 67.30 mg/kg, representing a decrease of 83.69%; the acid-soluble protein content reached 29.72%, an increase of 25.86 percentage points; the reducing sugar content was 19.49 mg/g, an increase of 13.89 mg/g; and the pH dropped by 1.59 units to 4.91. Analysis of the peptide molecular weight distribution revealed that 99.61% of the cottonseed peptides had a molecular weight below 10,000 Da, and 65.45% were below 1000 Da. The peptides also exhibited excellent antioxidant capacity. In conclusion, the microbial–enzymatic synergistic treatment significantly elevated the contents of acid-soluble protein, reducing sugars, and peptides, enhanced antioxidant capacity, and reduced both free gossypol content and pH, thereby effectively improving the nutritional quality of cottonseed protein.

## 1. Introduction

With the intensification of livestock production and the growing demand for efficient utilization of feed resources, the development of protein resources from agricultural by-products has become an important direction for the sustainable development of the feed industry. Cottonseed protein is a major by-product of cotton processing, with a crude protein content typically ranging from 40% to 60%, indicating considerable potential for feed application [1]. Moreover, owing to its high yield and wide availability, cottonseed protein is regarded as a plant protein resource with promising development prospects [2]. However, cottonseed protein contains anti-nutritional factors such as free gossypol and non-starch polysaccharides. Free gossypol not only impairs animal growth and reproductive performance but also binds to amino acids such as lysine, thereby reducing amino acid availability and overall protein nutritional value [3]. In addition, cottonseed protein raw materials from different sources and subjected to different processing methods vary substantially in protein content, fiber level, and nutrient digestibility, further limiting their application in animal diets. Therefore, pretreatment techniques such as fermentation and enzymatic hydrolysis, which can lower free gossypol content, modify protein structure, and improve nutrient digestibility, represent key approaches for enhancing the feeding value of cottonseed protein [4,5,6].

Studies have demonstrated that enzymatic hydrolysis of cottonseed protein can not only degrade anti-nutritional factors but also break down macromolecular substances into smaller, more digestible and absorbable molecules, thereby increasing nutrient digestibility and absorption [7]. Laccase catalyzes the intramolecular cyclization of the aldehyde and hydroxyl groups of gossypol to form an ortho-semiquinone radical and release hydroxyl radicals, leading to gossypol degradation [8]. Proteases improve feed efficiency by promoting protein digestion and increasing amino acid availability [9]. Among these, alkaline proteases achieve the highest degree of hydrolysis of non-degradable protein components in cottonseed meal and fermented cottonseed meal [10]. Microbial fermentation is also effective in enhancing the nutritional value of cottonseed protein. This is because during the fermentation process, there is an increase in the number of probiotics, as well as in nutrients such as peptides, amino acids, and microorganisms [11]. Studies have shown that yeast not only degrades anti-nutritional factors such as free gossypol but also increases cellular protein content, improves amino acid balance, and secretes various digestive enzymes [12,13]. Lactic acid bacteria can also degrade free gossypol and generate substantial amounts of volatile compounds such as lactic acid and acetic acid, thereby decreasing pH and improving feed palatability [14,15,16].

The microbial–enzymatic synergistic treatment organically combines fermentation and enzymatic hydrolysis, compensating for the limited efficacy and single-product shortcomings of either method alone. It fully exploits the multi-product benefits of probiotic fermentation and the specific, highly efficient action of enzymes, ultimately enhancing both the nutritional value and the feeding value of cottonseed protein. Moreover, treating cottonseed protein via fermentation, enzymatic hydrolysis, or microbial–enzymatic synergistic treatment can generate a series of bioactive peptides exhibiting ACE inhibitory activity [17], immunomodulatory activity [18], antimicrobial activity [19], and antioxidant activity [7].

Therefore, the present study focused on the anti-nutritional factor free gossypol and the indigestible macromolecular proteins and carbohydrates in cottonseed protein. A microbial–enzymatic synergistic system composed of laccase, alkaline protease, *Saccharomyces cerevisiae*, and *Lactobacillus acidophilus* was employed to treat cottonseed protein. The process conditions were optimized through single-factor experiments and response surface methodology to improve the nutritional value and bioactivity of the treated product. This work aims to provide a reference for alleviating the shortage of protein feed resources and for promoting the high-value utilization of unconventional feed resources through microbial–enzymatic synergistic treatment.

## 2. Materials and Methods

### 2.1. Materials

#### 2.1.1. Main Raw Materials

Cottonseed protein (60% crude protein content, 412.57 mg/kg free gossypol [FG] content) was purchased from Xinjiang Taikun Plant Protein Feed Co., Ltd. (Urumqi, China). Alkaline protease (enzyme activity ≥ 60,000 U/g) was obtained from Guangzhou Xintuoke Biotechnology Co., Ltd. (Guangzhou, China), and laccase (enzyme activity ≥ 10,000 U/g) was purchased from Cangzhou Xiasheng Enzyme Biotechnology Co., Ltd. (Cangzhou, China).

#### 2.1.2. Strains and Culture Media

*Saccharomyces cerevisiae* and *Lactobacillus acidophilus* were both provided by the laboratory of Xinjiang Agricultural University. The yeast culture medium consisted of the following components (g/L): peptone, 20; yeast extract, 10; glucose, 20. The medium was prepared at its natural pH and sterilized by autoclaving at 121 °C for 20 min. The lactic acid bacteria culture medium consisted of the following components (g/L): peptone, 10; beef extract, 5; yeast extract powder, 5; glucose, 20; KH_2_PO_4_, 2; triammonium citrate, 2; sodium acetate, 5; MgSO_4_, 0.2; MnSO_4_, 0.05; Tween 80, 1 mL. The medium was prepared at pH 6.8 ± 0.2 and sterilized by autoclaving at 121 °C for 20 min.

### 2.2. Methods

#### 2.2.1. Strain Activation

*Saccharomyces cerevisiae* was inoculated into liquid yeast culture medium and incubated at 30 °C for 48 h. *Lactobacillus acidophilus* was inoculated into liquid lactic acid bacteria culture medium and incubated at 37 °C for 24 h.

#### 2.2.2. Enzymatic Hydrolysis of Cottonseed Protein

First, 100 g of cottonseed protein was mixed thoroughly with laccase or alkaline protease, dispensed into 500 mL Erlenmeyer flasks, and subjected to enzymatic hydrolysis in a constant-temperature incubator with three replicates per treatment. The flasks were shaken once every 24 h. After hydrolysis, the samples were dried at 65 ± 5 °C to constant weight, ground, and passed through a 40-mesh sieve for determination of acid-soluble protein content. Four single-factor experiments were conducted to investigate the effects of the following variables on the acid-soluble protein content of cottonseed protein. (1) Temperature: the effect of temperature (25 °C, 30 °C, 35 °C, 40 °C, 45 °C, 50 °C) was examined under fixed conditions of 35% substrate moisture, hydrolysis time of 48 h, and 0.5% enzyme dosage. (2) Substrate moisture: The effect of substrate moisture (25%, 30%, 35%, 40%, 45%, 50%) was examined under fixed conditions of 35 °C, 48 h, and 0.5% enzyme dosage. (3) Hydrolysis time: The effect of hydrolysis time (0 h, 12 h, 24 h, 36 h, 48 h, 72 h, 96 h) was examined under fixed conditions of 35 °C, 35% substrate moisture, and 0.5% enzyme dosage. (4) Enzyme dosage: The effect of enzyme dosage (0%, 0.1%, 0.25%, 0.5%, 0.75%, 1%) was examined under fixed conditions of 35 °C, 35% substrate moisture, and 48 h. For laccase hydrolysis of cottonseed protein, free gossypol content was determined. Based on these experiments, the optimal conditions for the enzymatic hydrolysis of cottonseed protein by alkaline protease and laccase were established.

#### 2.2.3. Fermentation of Cottonseed Protein

Cottonseed protein (100 g) was thoroughly mixed with the bacterial inoculum, dispensed into 500 mL Erlenmeyer flasks, and subjected to fermentation in a constant-temperature incubator with three replicates per treatment. The flasks were shaken once every 24 h. After fermentation, the samples were dried at 65 ± 5 °C to constant weight, ground, and passed through an 80-mesh sieve for determination of acid-soluble protein content. Following the same four-variable design as described in Section 2.2.2, single-factor experiments were performed to investigate the effects of inoculum size (%), fermentation temperature (°C), substrate moisture (%), and fermentation time (h) on the acid-soluble protein content of cottonseed protein fermented by *S. cerevisiae* and *L. acidophilus*, respectively. The effect of *L. acidophilus* fermentation on pH under different conditions was also evaluated. Based on these experiments, the optimal conditions for the single-factor fermentation of cottonseed protein by *S. cerevisiae* and *L. acidophilus* were established.

#### 2.2.4. Microbial–Enzymatic Synergistic Treatment of Cottonseed Protein

Based on the results of the single-factor experiment, three factors—treatment temperature (A), substrate moisture content (B), and treatment time (C)—were selected. Using pH, acid-soluble protein, and free gossypol content as response variables, a three-factor, three-level response surface design was created using the BBD-RSM function in Design-Expert 13 software. Alkaline protease, laccase, *Saccharomyces cerevisiae*, and *Lactobacillus acidophilus* were added in equal proportions, each at a level of 1% of the cottonseed protein mass. The 1% enzyme addition referred to here denotes the percentage of the enzyme preparation (based on enzyme activity of ≥60,000 U/g for alkaline protease and ≥10,000 U/g for laccase) relative to the mass of cottonseed protein. The inoculation density for both *Saccharomyces cerevisiae* and *Lactobacillus acidophilus* was 1 × 10^8^ CFU/mL (based on live cultures), added at a volume ratio of 1% (i.e., 1 mL of culture per 100 g of cottonseed protein). These four components were added simultaneously to the cottonseed protein. Cottonseed protein (100 g) was weighed out and mixed thoroughly with the microbial cultures and enzymes. Then the mixture was transferred to 500 mL conical flasks and sealed with a sealing membrane. Three replicates were set up for each treatment, all placed in a constant-temperature incubator for fermentation, with shaking once every 24 h. Upon completion of the treatment, the samples were dried to constant weight at 65 ± 5 °C, ground, and sieved through an 80-mesh sieve, followed by determination of pH, acid-soluble protein, and free gossypol content. The experimental design is shown in Table 1.

#### 2.2.5. Preparation of Cottonseed Peptides

Cottonseed peptides were obtained by hydrolysis under the optimized conditions. The hydrolysate was centrifuged at 3500 rpm for 15 min, and the supernatant was collected, rapidly pre-frozen at −80 °C to a solid state, and finally subjected to vacuum freeze-drying. The operating parameters were as follows: freeze-drying area, 0.27 m^2^; cold trap temperature, −85 °C; vacuum degree, <9 Pa; and sublimation drying time, 12 h.

### 2.3. Analytical Determinations

#### 2.3.1. Determination of Nutritional Components

Determination of pH: A 10 g sample was placed in a 200 mL beaker, mixed with distilled water at a solid-to-water ratio of 1:9 (*w*/*v*), and stirred with a magnetic stirrer for 30 min. The pH was then measured using a Leici pH meter. Dry matter, crude protein, crude fat, crude ash, neutral detergent fiber, acid detergent fiber, calcium, and phosphorus contents were determined according to the methods described by Huang et al. [20]. Acid-soluble protein content was determined following the method of GB/T 22492-2008 [21]. Amino acid composition was analyzed using an amino acid analyzer (Artemis 6000, Techcomp, Shanghai, China) according to GB/T 5009.124-2016 [22]. Total sugar and reducing sugar contents were determined by the 3,5-dinitrosalicylic acid colorimetric method [23].

#### 2.3.2. Determination of Free Gossypol

Free gossypol content was determined according to GB/T 13086-2020 [24]. Principle: Free gossypol in the sample was extracted using a mixed solvent of isopropanol and n-hexane containing 3-amino-1-propanol. Following derivatization with aniline, the absorbance was measured at 440 nm, and the content was calculated using the mass absorption coefficient. Method: Reagent A was prepared by mixing isopropanol and n-hexane at a ratio of 6:4. Taking approximately 500 mL of Reagent A, 2 mL of 3-amino-1-propanol, 8 mL of glacial acetic acid, and 50 mL of water were added. The final volume was adjusted to 1000 mL with Reagent A to obtain Reagent B. Then, 1 g of cottonseed protein was weighed out, mixed with 50 mL of Reagent B, and shaken for 1 h, followed by filtration. Subsequently, 10 mL of the filtrate was taken, aniline was added, and the mixture was heated in a boiling water bath for 30 min. The solution was then diluted to volume and allowed to stand for 1 h. The absorbance was measured after zeroing the instrument with a reference solution. The free gossypol content (mg/kg) was calculated using Equation (1), where A is the corrected absorbance, m is the mass of the sample (g), V is the volume of the filtrate (mL), and L is the path length of the cuvette (cm). Precision requirements: When the content is <500 mg/kg, the difference between two determinations is ≤15% of the mean value; when 500–750 mg/kg, ≤75 mg/kg; when >750 mg/kg, ≤10%.

Free gossypol = (A × 1250 × 1000)/(a × m × v × l)(1)

#### 2.3.3. Determination of Relative Molecular Weight

The sample was placed in a 10 mL volumetric flask, diluted to volume with the mobile phase, ultrasonicated for 5 min, centrifuged, and filtered through a microporous membrane before injection. The molecular weight distribution of peptides was determined using a Waters 2695 high-performance liquid chromatography system equipped with a 487 UV detector (Waters Corporation, Milford, MA, USA) and Empower GPC software (v 3.0). A TSKgel 2000 SWxL column (300 mm × 7.8 mm, TOSOH Corporation, Tokyo, Japan) was used, with acetonitrile/water/trifluoroacetic acid (40/60/0.1, *v*/*v*/*v*) as the mobile phase at a flow rate of 0.5 mL/min, a column temperature of 30 °C, and a UV detection wavelength of 220 nm. The molecular weight calibration curve was constructed using cytochrome C (MW 12,384), aprotinin (MW 6500), bacitracin (MW 1422), glycine–glycine–tyrosine–arginine (MW 451), and glycine–glycine–glycine (MW 189) as standards.

#### 2.3.4. Determination of Antioxidant Capacity

DPPH radical scavenging capacity (A153-1-1, colorimetric method), hydroxyl radical (OH·) scavenging capacity (A018-1-1, colorimetric method), superoxide anion (O_2_·^−^) inhibition/production (A052-1-1, colorimetric method), and total antioxidant capacity (T-AOC, A015-1, colorimetric method) were all determined using commercial assay kits purchased from Nanjing Jiancheng Bioengineering Institute.

##### Determination of DPPH Radical Scavenging Capacity

First, 100 µL of cottonseed protein peptide solutions of different concentrations (0.5, 1, 2, 4, 8 mg/mL) were mixed thoroughly with 2.9 mL of a 0.1 mM DPPH methanol solution, then left to react in the dark for 30 min. The absorbance was measured at 517 nm for both the pure methanol solution and the sample reaction mixture. The DPPH radical scavenging rate was calculated using Equation (2), where A_control_ represents the absorbance of DPPH after the addition of pure methanol; A_sample_ represents the absorbance of DPPH after the addition of the cottonseed peptide solution; and A_blank_ represents the absorbance of the cottonseed peptide solution after the addition of methanol.

DPPH radical scavenging efficiency (%) = [l − (A_sample_ − A_blank_)/A_control_] × 100 (2)

##### Determination of Hydroxyl Radical Scavenging Capacity

The Fenton reaction generates hydroxyl radicals, with the amount of H_2_O_2_ being directly proportional to the amount of OH^−^ produced. Upon the addition of an electron acceptor, the reaction was visualized using Gries’ reagent, forming a red substance; the intensity of the color is directly proportional to the concentration of OH^−^. Definition: One unit of hydroxyl radical scavenging activity is defined as the amount of cottonseed peptide required to reduce the H_2_O_2_ concentration in the reaction system by 1 mmol/L after 1 min of reaction at 37 °C.

Hydroxyl radical scavenging capacity (U/mL) = (OD_control_ − OD_sample_)/(OD_standard_ − OD_blank_) × standard concentration (8.824 mmol/L) × 1/sample volume × sample dilution factor (3)

##### Determination of Superoxide Anion Radical Scavenging Activity

By simulating the reaction system between xanthine and xanthine oxidase in the body, superoxide anion radicals were generated; the addition of electron-donating substances and Gress’s coloring agent caused the reaction system to turn purplish-red, and its absorbance was measured using a spectrophotometer. When the sample under testing contained superoxide anion radical inhibitors, the absorbance of the test tube was lower than that of the control tube during the colorimetric analysis. Using vitamin C as a standard, the ability of the test item to inhibit superoxide anion radicals was calculated. Definition: In the reaction system, one activity unit is defined as the change in superoxide anion radical inhibition per liter of cottonseed meal oligopeptide after 40 min of reaction at 37 °C, equivalent to the inhibition achieved by 1 mg of vitamin C. Superoxide anion radical scavenging activity was calculated using Equation (4).

Anti-superoxide anion activity units (U/L) = (OD_control_ − OD_sample_)/(OD_control_ − OD_standard_) × standard concentration (0.15 mg/mL) × 1000 × sample dilution factor (4)

##### Determination of Total Antioxidant Capacity

Antioxidants can reduce Fe^3+^ to Fe^2+^, which can form stable complexes with phenanthroline derivatives; the antioxidant capacity can be determined via colorimetry. Definition: At 37 °C, one unit of total antioxidant capacity is defined as the amount of fermented cottonseed meal oligopeptide required to increase the optical density (OD) of the reaction system by 0.01 per microliter per minute. Total antioxidant capacity was calculated using Equation (5).

Total antioxidant capacity (U/mL) = (OD_reading_ − OD_blank_)/(sample volume × 0.01 × 30 min) × total reaction volume (3.7 mL) × sample dilution factor (5)

### 2.4. Statistical Analysis

Preliminary data organization and calculations were performed using Microsoft Excel 2021. Statistical analysis was conducted using IBM SPSS Statistics 29 software (IBM, Armonk, New York, NY, USA), employing an independent samples *t*-test. Results are expressed as the mean ± standard error (SEM). A *p*-value < 0.05 was considered statistically significant; a *p*-value > 0.05 was considered not statistically significant. Response surface design was analyzed using Design-Expert 13 software. Following data processing and analysis, graphs were plotted using Origin 2021 software.

## 3. Results

### 3.1. Optimization of the Cottonseed Protein Process

#### 3.1.1. Optimization of Laccase Hydrolysis of Cottonseed Protein

**Figure 1:**
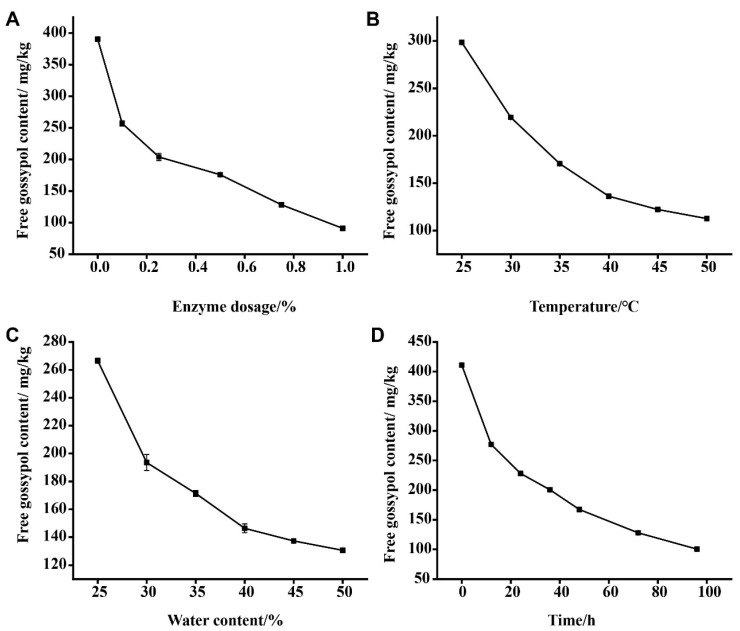
Effects of laccase on the free gossypol content of cottonseed protein. (**A**) enzyme dosage; (**B**) temperature; (**C**) water content; (**D**) time.

As shown in Figure 1, when the laccase dosage reached 1%, the free gossypol content decreased to 91.76 mg/kg. At an enzymatic hydrolysis temperature of 40 °C, the free gossypol content was reduced to 136.11 mg/kg, and increasing the temperature further resulted in no significant change in free gossypol content (*p* > 0.05). When the substrate moisture content was 40%, the free gossypol content was reduced to 147.05 mg/kg; beyond this moisture level, the decline leveled off. The lowest free gossypol content (100.32 mg/kg) was obtained when the hydrolysis time reached 96 h. Taken together, the optimal process parameters for laccase hydrolysis were determined as follows: enzyme dosage of 1%, temperature of 40 °C, 40% substrate moisture, and hydrolysis time of 96 h.

#### 3.1.2. Optimization of Alkaline Protease Hydrolysis of Cottonseed Protein

**Figure 2:**
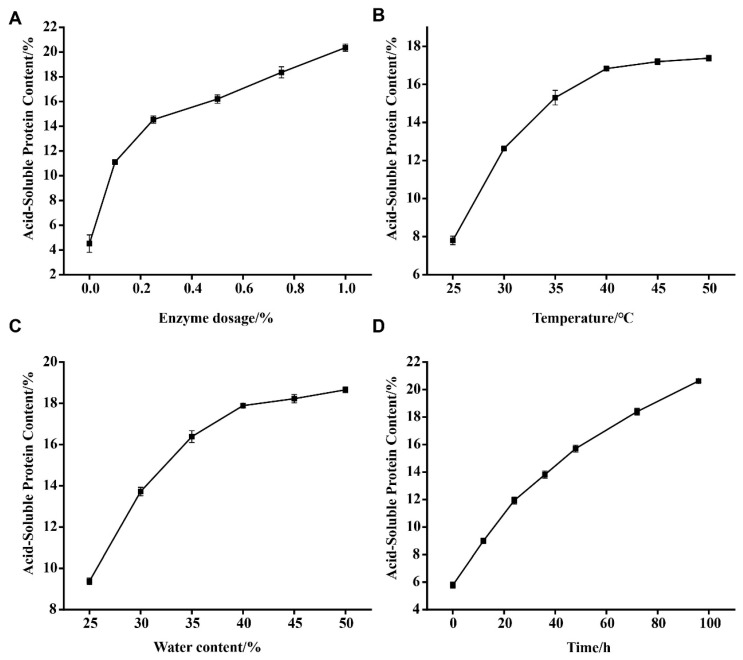
Effects of alkaline protease hydrolysis on the acid-soluble protein content of cottonseed protein. (**A**) enzyme dosage; (**B**) temperature; (**C**) water content; (**D**) time.

As shown in Figure 2, when the alkaline protease dosage reached 1%, the acid-soluble protein content reached 20.35%. At a temperature of 40 °C, the acid-soluble protein content was 16.83%, and increasing the temperature further did not result in significant changes (*p* > 0.05). When the substrate moisture was 40%, the acid-soluble protein content was 17.89%, and the increase became non-significant with further moisture elevation (*p* > 0.05). The maximum acid-soluble protein content (20.37%) was achieved when the hydrolysis time was extended to 96 h. In summary, the optimal process parameters for alkaline protease hydrolysis of cottonseed protein were enzyme dosage of 1%, temperature of 40 °C, 40% substrate moisture, and hydrolysis time of 96 h.

#### 3.1.3. Optimization of *Saccharomyces cerevisiae* Fermentation of Cottonseed Protein

**Figure 3:**
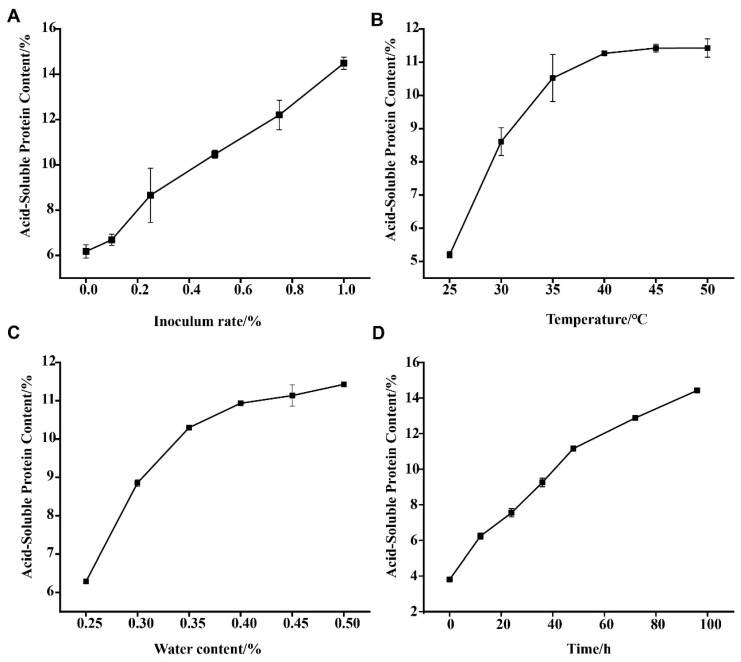
Effects of *Saccharomyces cerevisiae* fermentation on the acid-soluble protein content of cottonseed protein. (**A**) inoculum rate; (**B**) temperature; (**C**) water content; (**D**) time.

As shown in Figure 3, when the inoculum size of *S. cerevisiae* reached 1%, the acid-soluble protein content peaked at 14.49%. At a fermentation temperature of 35 °C, the acid-soluble protein content was 10.52%, and the curve plateaued with further temperature increases (*p* > 0.05). When the substrate moisture was 40%, the acid-soluble protein content was 10.93%, and further increases in moisture led to only slight, non-significant increments (*p* > 0.05). The highest acid-soluble protein content (14.42%) was observed at a fermentation time of 96 h. In summary, the optimal process parameters for *S. cerevisiae* fermentation of cottonseed protein were inoculum size of 1%, temperature of 35 °C, 40% substrate moisture, and fermentation time of 96 h.

#### 3.1.4. Optimization of *Lactobacillus acidophilus* Fermentation of Cottonseed Protein

**Figure 4:**
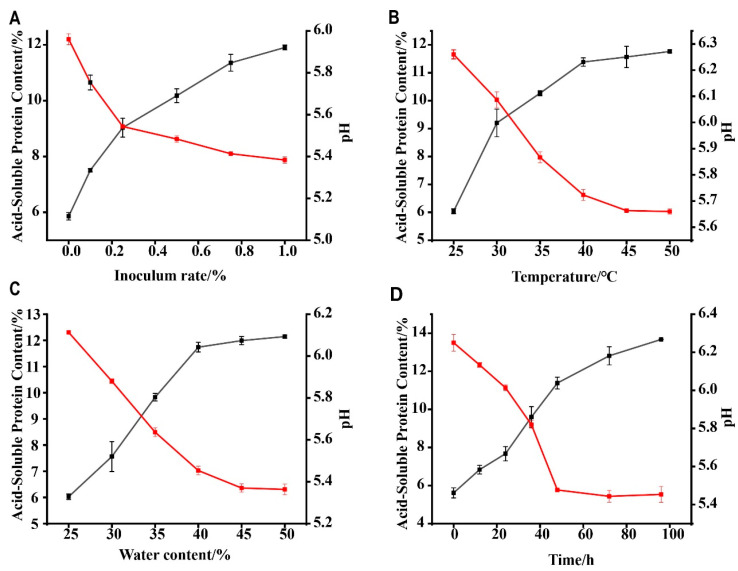
Effects of *Lactobacillus acidophilus* fermentation on the acid-soluble protein content and pH of cottonseed protein. (**A**) inoculum rate; (**B**) temperature; (**C**) water content; (**D**) time. In the figure, black represents acid-soluble protein content, whilst red represents pH.

As shown in Figure 4, when the inoculum size of *L. acidophilus* reached 1%, the acid-soluble protein content reached a maximum of 11.96%, accompanied by a decrease in pH to 5.38. When the temperature was raised to 40 °C, the acid-soluble protein content reached 11.38%, and further increases to 45 °C and 50 °C resulted in no significant differences (*p* > 0.05); the pH remained stable between 5.65 and 5.67 at temperatures above 40 °C. Therefore, 40 °C was selected as the optimal fermentation temperature. As the substrate moisture increased from 25% to 40%, the acid-soluble protein content rose rapidly from 6.03% to 11.74%, but the increase from 40% to 50% was modest (from 11.74% to 12.50%). The pH decreased from 6.11 to 5.37 as the moisture increased from 25% to 45%. Considering these results, 40% was chosen as the most appropriate moisture content. With prolonged fermentation time, the acid-soluble protein content gradually increased, reaching the highest value of 14.33% at 96 h. The pH of the material dropped to 5.48 at 48 h and remained relatively stable between 5.40 and 5.50 when the fermentation time was extended to 96 h. In summary, the optimal process parameters for *L. acidophilus* fermentation of cottonseed protein were inoculum size of 1%, temperature of 40 °C, 40% substrate moisture, and fermentation time of 96 h.

### 3.2. Verification of the Optimal Parameters for Single-Factor Treatments of Cottonseed Protein

As presented in Table 2, compared with the untreated cottonseed protein, alkaline protease treatment increased the acid-soluble protein content to 23.15% (an increase of 19.29 percentage points) and reduced the free gossypol content to 179.93 mg/kg, corresponding to a degradation rate of 56.39%. Laccase treatment increased the acid-soluble protein content to 7.43% (an increase of 3.57 percentage points) and decreased the free gossypol content to 88.73 mg/kg, with a degradation rate of 78.50%. *S. cerevisiae* fermentation increased the acid-soluble protein content to 16.81% (an increase of 12.95 percentage points) and reduced the free gossypol content to 160.59 mg/kg, corresponding to a degradation rate of 61.08%. *L. acidophilus* fermentation increased the acid-soluble protein content to 14.23% (an increase of 10.37 percentage points) and reduced the free gossypol content to 132.11 mg/kg, with a degradation rate of 67.98%.

### 3.3. Response Surface Experimental Results and Analysis of Variance

#### 3.3.1. Experimental Design and Results of Response Surface Methodology

Based on the results of the single-factor experiments, pH (d_1_), acid-soluble protein content (d_2_), and free gossypol content (d_3_) were selected as the response variables, with temperature (A), substrate moisture (B), and treatment time (C) as the three independent factors. A three-factor, three-level Box–Behnken design was employed for the optimization experiment, comprising a total of 17 experimental runs. An overall desirability (OD) index was calculated following the normalization method proposed by Hassan. For acid-soluble protein content, normalization was performed as normalized value = (X_i_ − X_min_)/(X_max_ − X_min_). Since lower values are desirable for pH and free gossypol content, these indicators were first positively transformed as: Y_i_ = X_max_ − X_i_, and subsequently normalized as normalized value = (Y_i_ − Y_min_)/(Y_max_ − Y_min_), where X_i_ is the measured value of the indicator, X_max_ and X_min_ are the maximum and minimum measured values, respectively; Y_i_ is the positively transformed value, and Y_max_ and Y_min_ are the maximum and minimum values of the positively transformed indicator. The overall desirability (OD) was calculated as OD = (d_1_ + d_2_ + d_3_)/3. The experimental results are presented in Table 3.

#### 3.3.2. Analysis of Variance for the Response Surface Model

The experimental data in Table 3 were fitted to a multiple regression model, yielding the following regression equation in terms of coded factors: OD = 0.7200 + 0.0952A + 0.2206B + 0.0521C + 0.0843AB + 0.0809AC + 0.0767BC − 0.0757A^2^ − 0.2022B^2^ − 0.1147C^2^. The analysis of variance (ANOVA) for the regression model is presented in Table 4. As shown in the ANOVA, the model was highly significant (*p* < 0.01), and the lack of fit was not significant (*p* = 0.0577 > 0.05), indicating that the model adequately described the data. The terms A, B, C, AB, AC, BC, A^2^, B^2^, and C^2^ in the model equation all exhibited significant (*p* < 0.05) or highly significant (*p* < 0.01) effects on OD. The F-value reflects the relative magnitude of a factor’s influence; a larger *F*-value corresponds to a stronger effect. Accordingly, the order of influence on OD was B (substrate moisture, %) > A (treatment temperature, °C) > C (treatment time, h). The coefficient of determination (*R*^2^) was 0.9695, demonstrating a high degree of model fit.

#### 3.3.3. Analysis of Interaction Effects Among Response Surface Factors

**Figure 5:**
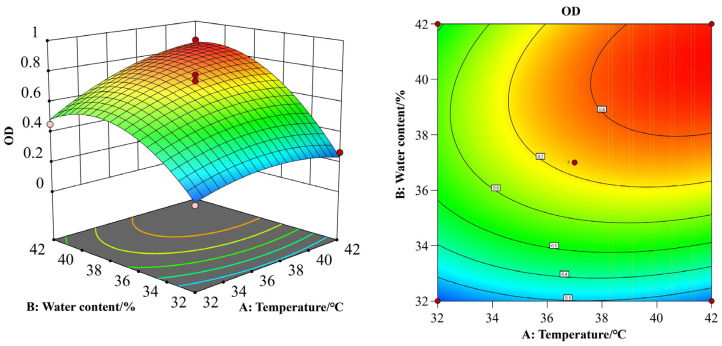
Three-dimensional response surface plot of the effect of treatment temperature and substrate moisture on OD. In the figure, the colour gradient indicates the magnitude of the OD values, with blue corresponding to lower OD values and red to higher OD values.

**Figure 6:**
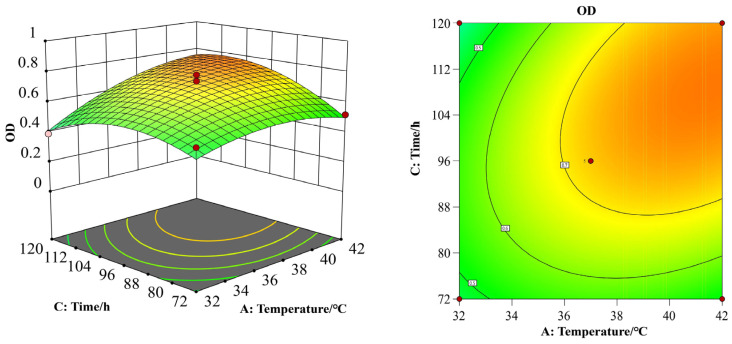
Three-dimensional response surface plot of the effects of treatment temperature and treatment time on OD. In the figure, the colour gradient indicates the magnitude of the OD values, with blue corresponding to lower OD values and red to higher OD values.

**Figure 7:**
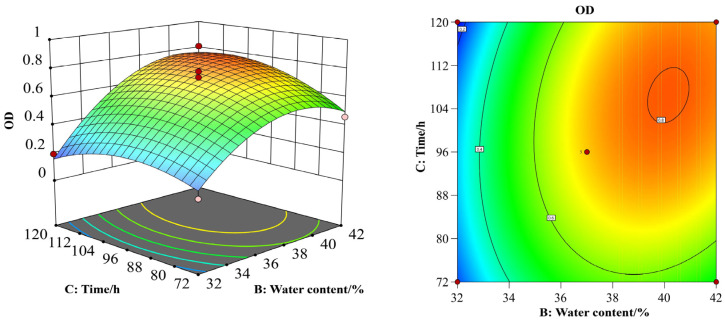
Three-dimensional response surface plot of the effects of treatment time and substrate moisture on OD. In the figure, the colour gradient indicates the magnitude of the OD values, with blue corresponding to lower OD values and red to higher OD values.

The strength of the interaction between two factors can be inferred from the density of the contour lines. When one factor is held at the center level, a stronger interaction between the other two factors is reflected by a steeper response surface and more closely spaced contour lines. As shown by the contour plots and 3D response surface plots in Figure 5, Figure 6 and Figure 7, the contour lines for the interactions between treatment temperature, treatment time, and substrate moisture tended to be elliptical, and the corresponding interaction surfaces were relatively steep, indicating strong effects on OD. These observations are consistent with the results of the analysis of variance.

#### 3.3.4. Verification Experiment for the Optimized Conditions Determined via Response Surface Methodology

As presented in Table 5, the response surface analysis identified the optimal conditions for the microbial–enzymatic synergistic treatment of cottonseed protein as follows: treatment temperature (A) of 37 °C, 37% substrate moisture (B), and treatment time (C) of 96 h. Under these conditions, the predicted overall desirability (OD) was 0.7200, with a predicted pH of 5.28, acid-soluble protein content of 29.95%, and free gossypol content of 67.23 mg/kg. Verification experiments performed in triplicate under these conditions yielded a pH of 4.91, an acid-soluble protein content of 29.72%, and a free gossypol content of 67.30 mg/kg. The experimental values were in close agreement with the predicted values, confirming the reliability of the optimized results.

### 3.4. Effects of Microbial–Enzymatic Synergistic Treatment of Cottonseed Protein

Based on the results described above, the nutritional profile of cottonseed protein treated with laccase, alkaline protease, *Saccharomyces cerevisiae*, and *Lactobacillus acidophilus* under the optimized conditions was determined. As shown in Table 6, compared with the untreated cottonseed protein group, the microbial–enzymatic synergistic treatment significantly increased the acid-soluble protein content (29.72%, an increase of 25.86 percentage points) and the reducing sugar content (19.49 mg/g, an increase of 13.89 mg/g) (*p* < 0.01). Meanwhile, the pH (4.91) and free gossypol content (67.30 mg/kg) were significantly decreased (*p* < 0.01); the pH dropped by 24.46%, and the free gossypol content was reduced by 83.69%. In contrast, no significant differences were observed between the two groups in dry matter, crude ash, crude protein, crude fat, calcium, phosphorus, neutral detergent fiber, or acid detergent fiber contents (*p* > 0.05).

### 3.5. Changes in Amino Acid Content Following Cotton Seed Protein

As shown in Table 7, compared with cottonseed protein, cottonseed peptides exhibited significantly higher contents of aspartic acid, threonine, glutamic acid, glycine, phenylalanine, and lysine (*p* < 0.05), and highly significantly higher contents of proline, alanine, valine, isoleucine, leucine, tyrosine, and histidine (*p* < 0.01). Moreover, the total amino acid content of cottonseed peptides was significantly higher than that of cottonseed protein (*p* < 0.05).

### 3.6. Changes in the Molecular Weight Distribution of Cotton Seed Protein

**Figure 8:**
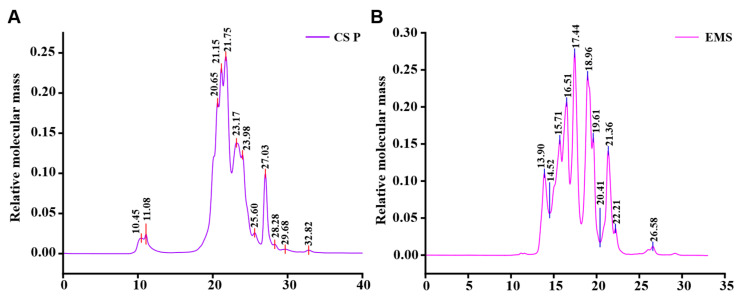
Comparison of relative molecular weight between cottonseed protein and cottonseed peptides. (**A**) cottonseed protein; (**B**) cottonseed peptides.

As shown in Table 8 and Figure 8, protein molecules with a molecular weight greater than 10,000 Da accounted for 58.06% of cottonseed protein, whereas in cottonseed peptides, this fraction represented only 0.39%. In the cottonseed peptides, 84.04% of the peptides had a molecular weight below 2000 Da, distributed as follows: 2000–1000 Da, 18.59%; 1000–500 Da, 21.24%; 500–180 Da, 23.59%; and below 180 Da, 20.62%. Moreover, both the weight-average molecular weight (Mw) and the number-average molecular weight (Mn) of cottonseed peptides were lower than those of cottonseed protein.

### 3.7. Antioxidant Activity of Cottonseed Peptides

**Figure 9:**
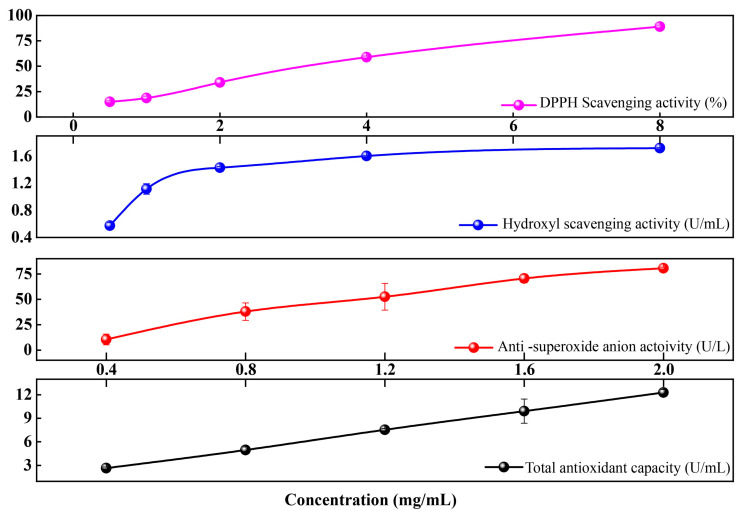
Effects of the microbial–enzymatic synergistic treatment on the antioxidant capacity of cottonseed protein.

As shown in Figure 9, across the concentration range of 0.5, 1.0, 2.0, 4.0, and 8.0 mg/mL, both the DPPH radical scavenging rate and the hydroxyl radical scavenging capacity of cottonseed peptides increased with increasing concentration. The DPPH scavenging rate displayed a continuously rising trend, reaching a maximum of 88.96% at 8.0 mg/mL. The hydroxyl radical scavenging capacity tended to plateau after the concentration reached 2.0 mg/mL, attaining a maximum value of 1.72 U/mL at 8.0 mg/mL. Within the concentration range of 0.4, 0.8, 1.2, 1.6, and 2.0 mg/mL, both the superoxide anion scavenging capacity and the total antioxidant capacity (T-AOC) of cottonseed peptides increased with increasing concentration, without showing an obvious plateau. At 2.0 mg/mL, the superoxide anion scavenging capacity reached its highest level of 80.59 U/L, and the total antioxidant capacity peaked at 12.29 U/mL.

## 4. Discussion

### 4.1. Effects of Enzymatic Hydrolysis on the Nutritional Value of Cottonseed Protein

Cottonseed protein is a by-product of cottonseed processing with high protein content, wide availability, and considerable feeding potential; however, its utilization has long been constrained by the presence of free gossypol. Gossypol is a tetraterpenoid aldehyde secondary metabolite that exists in the pigment glands of cotton roots, stems, and seeds, with the highest abundance in cottonseeds [25]. Gossypol occurs primarily in free and bound forms. Free gossypol, which contains highly reactive aldehyde and phenolic hydroxyl groups, readily interacts with proteins, amino acids, and metal ions, thereby adversely affecting animal physiological metabolism and production performance [26]. Therefore, reducing the free gossypol content is a prerequisite for the high-value utilization of cottonseed protein. The results of the present study demonstrate that laccase treatment significantly reduced the free gossypol content in cottonseed protein. Under the conditions of 1% enzyme dosage, 40 °C, 40% substrate moisture, and 96 h of treatment, the free gossypol content decreased from 412.57 mg/kg to 100.35 mg/kg, corresponding to a degradation rate of 75.7%. This finding indicates that laccase can effectively degrade anti-nutritional factors in cottonseed protein. Previous studies have shown that laccase, as a copper-containing polyphenol oxidase, catalyzes the oxidation of phenolic substrates and can reduce the toxicity of gossypol by inducing intramolecular cyclization or structural transformation [8,27]. Zhang et al. [28] further reported that heterologously expressed CotA laccase achieved 100% degradation of free gossypol within 1 h at 37 °C and pH 7.0 without the addition of redox mediators, and that the addition of CotA to defatted cottonseed meal resulted in 87–98% degradation of free gossypol within 2 h. Beyond reducing free gossypol, improving the digestibility and small peptide content of cottonseed protein represents another objective for enhancing its nutritional value. Cottonseed protein contains a substantial amount of macromolecular proteins that are tightly structured in their native state, which may limit the accessibility of endogenous digestive enzymes to peptide bonds. Alkaline protease, an important member of the serine protease family, efficiently hydrolyzes peptide bonds and recognizes the carboxyl termini of hydrophobic amino acid residues [29,30]. Its catalytic activity relies on the Ser-His-Asp triad in the active center, which determines its catalytic efficiency toward substrates [31]. Acid-soluble protein was selected as an indicator of protein hydrolysis because it mainly represents small peptides and soluble nitrogenous fractions that remain soluble after acid precipitation of macromolecular proteins. Therefore, it reflects the conversion of macromolecular or insoluble protein fractions into soluble low-molecular-weight nitrogenous compounds during enzymatic hydrolysis and fermentation [32]. In this study, alkaline protease hydrolysis of cottonseed protein resulted in a gradual increase in acid-soluble protein content with increasing enzyme dosage, temperature, substrate moisture, and treatment time. The optimal conditions were identified as enzyme dosage of 1%, temperature of 40 °C, 40% substrate moisture, and treatment time of 96 h, under which the acid-soluble protein content of cottonseed protein reached 23.15%, indicating that the protein structure underwent substantial degradation. Related studies have also demonstrated that alkaline protease can degrade macromolecular proteins in cottonseed protein into small peptides or amino acids, thereby improving digestibility and absorption [33]. Moreover, enzymatic hydrolysis can produce low-molecular-weight peptide fractions with antioxidant, antimicrobial, or angiotensin-converting enzyme (ACE) inhibitory activities. Song et al. [34] prepared cottonseed protein hydrolysates using Alcalase and isolated fractions composed of low-molecular-weight peptides, all of which exhibited DPPH and ABTS radical scavenging capacities. de Oliveira Filho et al. [19] compared the hydrolysis of cottonseed protein by Alcalase, Neutrase, and Flavourzyme and found that the hydrolysates displayed stronger antioxidant activity than the unhydrolyzed protein, along with ACE inhibitory and antimicrobial activities; among these, the Alcalase hydrolysate exhibited relatively high ACE inhibitory activity. Wang et al. [7] prepared and identified antioxidant peptides from cottonseed protein hydrolysates and reported that the fraction with a molecular weight below 3 kDa possessed strong DPPH, ABTS, and hydroxyl radical scavenging capacities as well as ferrous ion chelating ability. Taken together, laccase mainly reduces anti-nutritional factors through oxidative transformation or degradation of free gossypol, whereas alkaline protease primarily increases the proportion of acid-soluble protein and small peptides by hydrolyzing macromolecular proteins. These two approaches address the objectives of detoxification and protein pre-digestion, respectively, and provide a theoretical foundation for the subsequent microbial–enzymatic synergistic treatment.

### 4.2. Effects of Fermentation on the Nutritional Value of Cottonseed Protein

Microbial fermentation technology enables the directional conversion of raw materials into functional products and their components through the precise regulation of culture environmental parameters. A large body of research has demonstrated that microbial fermentation can effectively degrade free gossypol and macromolecular proteins in cottonseed meal, thereby improving its nutritional profile and palatability [16]. The microorganisms commonly used in cottonseed protein fermentation include Bacillus spp., lactic acid bacteria, yeasts, and Aspergillus spp. For example, after solid-state fermentation of cottonseed meal with Bacillus subtilis for 48 h, the free gossypol content decreased from 0.82 g/kg to 0.21 g/kg, while the contents of crude protein and certain amino acids increased; the fermented product could partially replace soybean meal in broiler diets [35]. Yeasts are widely distributed in nature and amenable to large-scale cultivation, and they are extensively used to improve the crude protein and mineral content of plant-based feedstuffs and to reduce anti-nutritional factors through fermentation [36]. In the present study, cottonseed protein was fermented with *Saccharomyces cerevisiae* under the conditions of 1% inoculum size, 35 °C, 40% substrate moisture, and 96 h of fermentation. Under these conditions, the acid-soluble protein content increased from 3.86% to 16.81% (an increase of 12.95 percentage points), and the free gossypol content was reduced from 412.57 mg/kg to 160.59 mg/kg, corresponding to a degradation rate of 61.08%. During solid-state fermentation, yeasts can utilize a portion of carbon and soluble nitrogen sources for growth, and the proteases, peptidases, and other metabolic enzymes they secrete may participate in protein degradation and gossypol transformation. Mageshwaran et al. [13] used a mixed culture of Candida tropicalis and *S. cerevisiae* to ferment cottonseed meal and found that the free gossypol content was reduced by 60–80%, the crude protein content increased by 4–12%, and the fiber content decreased by 3–11%, indicating that yeast-mediated solid-state fermentation can effectively improve the safety and nutritional quality of cottonseed by-products. Lin et al. [15] employed a probiotic combination containing *S. cerevisiae*, Enterococcus faecium, and Lactobacillus plantarum for fermentation and observed that fermentation increased the crude protein, free amino acid, and total phosphorus contents of defatted cottonseed meal and improved its overall nutritional quality. Lactic acid bacteria (LAB) are also commonly used as functional microorganisms in fermented feed. As heterotrophic microorganisms, LAB are characterized by their ability to convert carbohydrates into lactic acid and other organic acids. These organic acids can modulate the intestinal microbiota of the host, improve the microecological environment, and enhance the immune function of the organism [37]. Yusuf et al. [38] reported that fermentation with LAB and yeasts decreased the free gossypol content and increased the crude protein content. Wang et al. [14] isolated a strain of Lactobacillus agilis WWK129 from the rumen of dairy cows. When incubated in vitro at 39 °C for 5 days with a 5% inoculum, this strain achieved a gossypol degradation rate of 83%, and the crude protein and essential amino acid contents increased concomitantly with lactic acid production. *Lactobacillus acidophilus*, a member of the genus Lactobacillus within the LAB group, is a recognized and directly absorbable probiotic. As a strain used in fermented feed, it can effectively lower pH and improve feed value [39]. In this study, *L. acidophilus* was used to ferment cottonseed protein under the conditions of 1% inoculum size, 40 °C, 40% substrate moisture, and 96 h of fermentation. The acid-soluble protein content increased from 3.86% to 14.23% (an increase of 10.37 percentage points), and the free gossypol content was reduced to 132.11 mg/kg, with a degradation rate of 67.97%. Moreover, microbial fermentation not only reduces the free gossypol content in cottonseed meal and increases the contents of water-soluble proteins and peptides, but the bioactive peptides prepared from fermented cottonseed meal also exhibit strong DPPH radical scavenging activity, hydroxyl radical scavenging activity, and reducing power [40,41]. This may be attributed to the combined effects of microbial acid production lowering pH and inhibiting the growth of spoilage organisms, the secretion of enzyme systems that degrade gossypol, and the conversion of macromolecular proteins into small peptides and free amino acids by proteases/peptidases, thereby collectively enhancing the safety, palatability, and nutritional utilization value of cottonseed protein.

### 4.3. Effects of Microbial–Enzymatic Synergistic Treatment on the Nutritional Value of Cottonseed Protein

Microbial–enzymatic synergistic treatment refers to the combined application of microorganisms and enzyme preparations, which fully exploits the dual advantages of the multi-product benefits of probiotic fermentation and the specific, highly efficient action of enzymes. Compared with single microbial fermentation or enzymatic hydrolysis, microbial–enzymatic synergistic treatment can accelerate the degradation of macromolecular substances in feed, thereby shortening the fermentation period and improving fermentation efficiency [42]. In the present study, the optimal conditions for the microbial–enzymatic synergistic treatment of cottonseed protein were determined via response surface optimization as follows: microbial and enzyme dosages each at 1% (*w*/*w*), temperature of 37 °C, 37% substrate moisture, and treatment time of 96 h. Under these conditions, the free gossypol content was reduced to 67.30 mg/kg, the acid-soluble protein content increased to 29.72%, the reducing sugar content increased to 19.49 mg/g, and the pH decreased to 4.91. When compared with the results of the individual enzymatic hydrolysis and fermentation treatments conducted earlier in this study, these findings indicate that the microbial–enzymatic synergistic treatment was superior to the single-treatment approaches in terms of both detoxification and protein pre-digestion. Ni et al. [43] used Bacillus subtilis, kefir consortium, and alkaline protease for the synergistic treatment of cottonseed meal and reported that the free gossypol removal rate reached 78%, the total soluble protein content increased to 58.11%, the angiotensin I-converting enzyme (ACE) inhibitory activity reached 86.72%, and the antioxidant activity reached 87.51%, accompanied by increased contents of carboxylic acids, organic oxides, benzene derivatives, fatty acids, and their derivatives. Lv et al. [44,45] employed Lactobacillus mucosae LLK-XR1 and acid protease for the synergistic treatment of cottonseed meal and found that the free gossypol removal rate reached 85.63%, the small peptide content increased to 46.25%, and the contents of free amino acids, soluble proteins, and partial available energy for animals were also elevated. Microbial–enzymatic synergistic treatment can not only improve the nutritional value of cottonseed protein but also promote the release of antioxidant bioactive peptides. Low-molecular-weight peptides generally exhibit good water solubility, diffusibility, and potential for digestion and absorption, and are more likely to expose active sites of hydrophobic amino acids, aromatic amino acids, and sulfur-containing amino acids, thereby enhancing free radical scavenging and antioxidant capacity [46,47]. The present study demonstrated that after microbial–enzymatic synergistic treatment, 99.61% of the resulting cottonseed peptides had a molecular weight below 10,000 Da, and 65.45% were below 1000 Da. Moreover, the total amino acid content was elevated, and the DPPH radical scavenging rate, hydroxyl radical scavenging capacity, superoxide anion scavenging capacity, and total antioxidant capacity were all enhanced. Wang et al. [48] co-fermented cottonseed meal with Bacillus subtilis natto N-2 and Meyerozyma guilliermondii WST-M1 and successfully prepared low-gossypol cottonseed protein peptides with a total protein content of 59.47% and a high proportion of small peptides (91.93% below 3000 Da, of which 78.24% were below 1000 Da), while simultaneously reducing the total gossypol and free gossypol contents. In the present experimental system, alkaline protease and laccase can rapidly disrupt gossypol-binding structures and the spatial conformation of macromolecular proteins, releasing soluble nitrogen sources that are promptly utilized by yeast/lactic acid bacteria for growth and acid production. The accumulation of organic acids, in turn, promotes enzyme activity and microbial metabolism. This simultaneous addition of strains and enzyme preparations is similar to the co-fermentation mode adopted by Ni et al. [43], but differs distinctly from the stepwise sequential treatment employed by Wang et al. [48]; nevertheless, the free gossypol removal rate and the yield of small peptides obtained in this study confirm the feasibility of the present method. Overall, the combined use of enzymes and microorganisms can reduce free gossypol whilst increasing acid-soluble protein content and the production of small-molecule peptides, making it one of the approaches for the high-value utilization of cottonseed protein.

## 5. Conclusions

Under conditions where the addition of both microbial enzymes was 1%, the temperature was 37 °C, the substrate moisture content was 37%, and the reaction time was 96 h, the free gossypol content decreased to 67.48 mg/kg, the pH decreased to 4.91, and the acid-soluble protein content increased to 29.72%. In addition, 99.61% of the cottonseed peptides had a molecular weight of less than 10,000 Da, whilst their DPPH radical scavenging rate, hydroxyl radical scavenging capacity, superoxide anion scavenging capacity, and total antioxidant capacity were all enhanced. The next phase of this study will involve a systematic evaluation of the palatability, storage stability, and safety of the treated products, as well as their impact on animal growth performance, thereby providing a basis for their application as a feed protein resource.

## Figures and Tables

**Table 1:** Factors and levels for the response surface experiment.

Level	Factor		
	A/°C	B/%	C/h
−1	32	32	72
0	37	37	96
1	42	42	120

**Table 2:** Verification of the optimal parameters for single-factor treatments of cottonseed protein.

Items	Acid-Soluble Protein (%)	Free Gossypol (mg/kg)
Cottonseed protein	3.86 ± 0.47	412.57 ± 9.22
Alkaline protease	23.15 ± 0.59	179.93 ± 1.72
Laccase	7.43 ± 0.22	88.73 ± 1.21
*Saccharomyces cerevisiae*	16.81 ± 0.18	160.59 ± 1.44
*Lactobacillus acidophilus*	14.23 ± 0.18	132.11 ± 0.95

**Table 3:** Experimental design and results of the response surface experiment.

Run	A/°C	B/%	C/h	pH	Acid-Soluble Protein (%)	Free Gossypol (mg/kg)	OD
1	32	32	96	5.36	21.66	93.75	0.1925
2	42	32	96	5.80	23.51	75.29	0.2670
3	32	42	96	4.95	25.75	95.63	0.4485
4	42	42	96	4.77	30.71	73.45	0.8603
5	32	37	72	5.15	25.70	77.60	0.5325
6	42	37	72	5.25	25.52	75.90	0.5082
7	32	37	120	5.17	22.58	81.30	0.3892
8	42	37	120	5.22	31.47	79.67	0.6886
9	37	32	72	5.71	22.98	89.94	0.1559
10	37	42	72	5.41	27.49	83.45	0.4604
11	37	32	120	5.68	26.18	99.76	0.1924
12	37	42	120	4.79	30.17	77.26	0.8037
13	37	37	96	5.26	29.27	59.80	0.7667
14	37	37	96	5.27	30.56	67.47	0.7433
15	37	37	96	5.32	30.55	69.62	0.7088
16	37	37	96	5.29	29.71	69.87	0.6879

**Table 4:** Analysis of variance for the regression model.

Source	Sum of Squares	df	Mean Square	F-Value	*p*-Value
Model	0.8371	9	0.093	25.62	0.0002
A	0.0725	1	0.0725	19.96	0.0029
B	0.3894	1	0.3894	107.28	<0.0001
C	0.0217	1	0.0217	5.98	0.0443
AB	0.0284	1	0.0284	7.83	0.0266
AC	0.0262	1	0.0262	7.22	0.0313
BC	0.0235	1	0.0235	6.48	0.0383
A2	0.0241	1	0.0241	6.64	0.0366
B2	0.1722	1	0.1722	47.42	0.0002
C2	0.0554	1	0.0554	15.25	0.0059
Residual	0.0254	7	0.0036		
Lack of Fit	0.0208	3	0.0069	6.03	0.0577
Pure Error	0.0046	4	0.0012		
Cor Total	0.8625	16			

**Table 5:** Results of the response surface verification experiment.

Items	pH	Acid-Soluble Protein (%)	Free Gossypol (mg/kg)
1	4.97	29.86	63.98
2	4.90	29.70	69.97
3	4.86	29.59	67.94
Mean	4.91	29.72	67.30

**Table 6:** Effects of microbial–enzymatic synergistic treatment on the nutritional level of cottonseed protein.

Items	Cottonseed Protein	Microbial–Enzymatic Synergistic Treatment	SEM	*p*-Value
DM (%)	92.40	92.76	0.197	0.208
Ash (%)	6.99	6.95	0.207	0.836
CP (%)	60.21	60.62	0.135	0.080
EE (%)	1.32	1.43	0.249	0.682
Ca (%)	0.49	0.49	0.004	0.928
Ph (%)	0.83	0.84	0.027	0.779
NDF (%)	21.21	21.18	0.450	0.951
ADF (%)	8.18	8.21	0.285	0.913
Acid-soluble protein (%)	3.86 ^Bb^	29.72 ^Aa^	0.282	<0.01
Total sugar (mg/g)	230.01 ^Aa^	217.25 ^Bb^	0.907	0.009
Reducing sugar (mg/g)	5.60 ^Bb^	19.49 ^Aa^	0.866	<0.01
pH	6.50 ^Aa^	4.91 ^Bb^	0.063	<0.01
Free gossypol (mg/kg)	412.57 ^Aa^	67.30 ^Bb^	5.609	<0.01

Note: Within-group data: Different uppercase letters indicate extremely significant differences (*p* < 0.01); different lowercase letters indicate significant differences (*p* < 0.05); identical letters or no letters indicate no significant differences (*p* > 0.05). SEM, standard error of the mean.

**Table 7:** Comparison of amino acid contents between cottonseed protein and cottonseed peptides.

Items	Cottonseed Protein	Cottonseed Peptides	SEM	*p*-Value
Aspartic acid	5.78 ^b^	6.76 ^a^	0.334	0.042
Threonine	2.06 ^b^	2.39 ^a^	0.113	0.044
Serine	2.97	3.32	0.177	0.121
Glutamic acid	12.80 ^b^	15.15 ^a^	0.630	0.020
Proline	2.02 ^Bb^	2.59 ^Aa^	0.046	<001
Glycine	2.58 ^b^	3.15 ^a^	0.129	0.012
Alanine	2.46 ^Bb^	3.20 ^Aa^	0.120	0.004
Cysteine	0.33	0.34	0.031	0.807
Valine	2.51 ^Bb^	3.27 ^Aa^	0.084	<001
Methionine	0.73	1.07	0.197	0.223
Isoleucine	1.84 ^Bb^	2.39 ^Aa^	0.045	<001
Leucine	3.53 ^Bb^	4.23 ^Aa^	0.106	0.003
Tyrosine	2.02 ^Bb^	2.35 ^Aa^	0.054	0.004
Phenylalanine	3.25 ^b^	3.77 ^a^	0.119	0.012
Histidine	2.35 ^Bb^	2.82 ^Aa^	0.068	0.002
Lysine	2.62 ^b^	3.04 ^a^	0.101	0.015
Arginine	7.59	7.49	0.447	0.831
Total amino acids	57.46 ^b^	67.33 ^a^	2.490	0.017

Note: Within-group data: Different uppercase letters indicate extremely significant differences (*p* < 0.01); different lowercase letters indicate significant differences (*p* < 0.05); identical letters or no letters indicate no significant differences (*p* > 0.05). SEM, standard error of the mean.

**Table 8:** Comparison of relative molecular weight between cottonseed protein and cottonseed peptides.

Cottonseed Protein				Cottonseed Peptides			
Molecular Weight Range	Mn	Mw	Peak Area (%)	Molecular Weight Range	Mn	Mw	Peak Area (%)
>100w	1,305,733	1,352,074	2.86	>10,000	17,602	19,813	0.39
100w~50w	779,183	807,697	1.22	10,000~5000	5678	5767	2.42
50w~20w	321,928	347,870	0.3	5000~3000	3967	4048	7.21
20w~10w	136,215	141,771	0.24	3000~2000	2381	2412	5.94
10w~5w	62,627	65,008	0.7	2000~1000	1345	1401	18.59
5w~2w	25,145	26,258	15.91	1000~500	667	695	21.24
2w~1w	14,022	14,529	36.82	500~180	259	280	23.59
<10,000	2250	4998	41.94	<180	31	86	20.62

Mn = number-average molecular weight; Mw = weight-average molecular weight.

## Data Availability

The original contributions presented in this study are included in the article. Further inquiries can be directed to the corresponding author.

## Abbreviations

The following abbreviations are used in this manuscript:

FG	Free gossypol
OD	Overall desirability
BBD	Box–Behnken design
RSM	Response surface methodology
ANOVA	Analysis of variance
SEM	Standard error of the mean
DM	Dry matter
CP	Crude protein
EE	Ether extract
Ca	Calcium
P	Phosphorus
NDF	Neutral detergent fiber
ADF	Acid detergent fiber
Mn	Number average molecular weight
Mw	Weight average molecular weight
DPPH	2,2-Diphenyl-1-picrylhydrazyl
T-AOC	Total antioxidant capacity
ACE	Angiotensin-converting enzyme
TFA	Trifluoroacetic acid
